# Exploring challenges of rcts in the emergency and critical care setting

**DOI:** 10.1186/2197-425X-3-S1-A765

**Published:** 2015-10-01

**Authors:** P Mouncey, S Power, DA Harrison, SE Harvey, KM Rowan

**Affiliations:** ICNARC, Clinical Trials Unit, London, United Kingdom

## Introduction

Approximately 50% of randomised controlled trials (RCTs) fail to recruit to time and target. RCTs in the emergency and critical care settings pose additional challenges including time of presentation of potentially eligible patients and the time required to formally recruit patients.

## Objectives

To describe the screening and recruitment patterns in RCTs in an emergency/critical care setting in the National Health Service (NHS) in England and to explore the impact that time taken to consent patients may have on the delivery of an early intervention.

## Methods

We recently completed two large multicentre RCTs in the emergency and critical care setting. Both RCTs were evaluating the delivery of an early intervention - early, goal-directed therapy (a resuscitation protocol) (n=1260 patients in 56 sites)[1] and early nutritional support (n=2400 patients in 33 sites)[2]. As well as collecting data for the evaluation, we also collected data around screening, recruitment and timing.

## Results

In both trials, due to the emergency/critical care setting, recruitment rates were lower - with eligible patients missed - at nights and at weekends with an absence of study “delegated” staff available often cited as the reason. In both trials, 81% of patients were recruited Monday to Friday 08:00 to 19:59. In the trial on resuscitation, the time taken to formally recruit patients impacted on the early nature of the intervention (time from meeting the inclusion criteria to randomisation was on average 1.1 hours).

## Conclusions

Until research infrastructure can be delivered 24/7 in the NHS in England, RCTs in the emergency and critical care setting will struggle to deliver to time and target. The use of deferred consenting procedures for time-sensitive interventions warrants further debate.

## Grant Acknowledgment

Both RCTs were funded by the National Institute for Health Research Health Technology Assessment Programme (07/37/47; 07/52/03).
